# A Transcriptomics Approach To Unveiling the Mechanisms of *In Vitro* Evolution towards Fluconazole Resistance of a *Candida glabrata* Clinical Isolate

**DOI:** 10.1128/AAC.00995-18

**Published:** 2018-12-21

**Authors:** Mafalda Cavalheiro, Catarina Costa, Ana Silva-Dias, Isabel M. Miranda, Can Wang, Pedro Pais, Sandra N. Pinto, Dalila Mil-Homens, Michiyo Sato-Okamoto, Azusa Takahashi-Nakaguchi, Raquel M. Silva, Nuno P. Mira, Arsénio M. Fialho, Hiroji Chibana, Acácio G. Rodrigues, Geraldine Butler, Miguel C. Teixeira

**Affiliations:** a Department of Bioengineering, Instituto Superior Técnico, Universidade de Lisboa, Lisbon, Portugal; b iBB—Institute for Bioengineering and Biosciences, Biological Sciences Research Group, Instituto Superior Técnico, Universidade de Lisboa, Lisbon, Portugal; c Department of Pathology, Division of Microbiology, Faculty of Medicine, University of Porto, Porto, Portugal; d CINTESIS—Center for Health Technology and Services Research, Faculty of Medicine, University of Porto, Porto, Portugal; e School of Biomolecular and Biomedical Science, Conway Institute, University College Dublin, Belfield, Dublin, Ireland; f Centro de Química-Física Molecular, Instituto Superior Técnico, Universidade de Lisboa, Lisbon, Portugal; g Medical Mycology Research Center, Chiba University, Chiba, Japan; h Department of Medical Sciences, iBiMED and IEETA, University of Aveiro, Aveiro, Portugal

**Keywords:** *Candida glabrata*, Epa3, azole drug resistance, evolution, transcriptomics

## Abstract

Candida glabrata is an emerging fungal pathogen. Its increased prevalence is associated with its ability to rapidly develop antifungal drug resistance, particularly to azoles. In order to unravel new molecular mechanisms behind azole resistance, a transcriptomics analysis of the evolution of a C. glabrata clinical isolate (isolate 044) from azole susceptibility to posaconazole resistance (21st day), clotrimazole resistance (31st day), and fluconazole and voriconazole resistance (45th day), induced by longstanding incubation with fluconazole, was carried out. All the evolved strains were found to accumulate lower concentrations of azole drugs than the parental strain, while the ergosterol concentration remained mostly constant. However, only the population displaying resistance to all azoles was found to have a gain-of-function mutation in the C. glabrata *PDR1* gene, leading to the upregulation of genes encoding multidrug resistance transporters. Intermediate strains, exhibiting posaconazole/clotrimazole resistance and increased fluconazole/voriconazole MIC levels, were found to display alternative ways to resist azole drugs. Particularly, posaconazole/clotrimazole resistance after 31 days was correlated with increased expression of adhesin genes. This finding led us to identify the Epa3 adhesin as a new determinant of azole resistance. Besides being required for biofilm formation, Epa3 expression was found to decrease the intracellular accumulation of azole antifungal drugs. Altogether, this work provides a glimpse of the transcriptomics evolution of a C. glabrata population toward multiazole resistance, highlighting the multifactorial nature of the acquisition of azole resistance and pointing out a new player in azole resistance.

## Abstract (precis)

Candida glabrata is an emerging fungal pathogen. Its increased prevalence is associated with its ability to rapidly develop antifungal drug resistance, particularly to azoles.

## INTRODUCTION

Candida glabrata is today the second or third most common cause of candidiasis, most likely because of its resistance to antifungal drugs, particularly azole drugs, which are used as prophylaxis and first- or second-line therapy (1, 2). Early azole formulations, such as the imidazoles miconazole, clotrimazole, and ketoconazole, are frequently used for the treatment of fungal mucocutaneous infections, even though they exhibit some toxicity in the treatment of systemic infections (3). The triazole drug fluconazole has been extensively used in prophylaxis and in the treatment of candidiasis, favoring the increase in drug-resistant C. glabrata infections (1, 4). Triazole drugs are significantly safer and more tolerable in systemic therapy than imidazoles (5), while newer triazoles, such as posaconazole and voriconazole, exhibit broader-spectrum and more-potent activity than fluconazole (6).

The most common cause of clinically acquired azole resistance is the upregulation of genes encoding drug efflux pumps from the ATP-binding cassette (ABC) superfamily and the major facilitator superfamily (MFS). One particular ABC transporter, C. glabrata Cdr1 (CgCdr1), is often involved in the acquisition of fluconazole resistance in C. glabrata isolates (7, 8). Additionally, CgCdr2/Phd1 and CgSnq2, two other ABC drug efflux pumps, have also been associated with fluconazole resistance in C. glabrata, their overexpression often resulting from the acquisition of gain-of-function (GOF) mutations in the *CgPDR1* gene (8, 9). Several MFS multidrug transporters have also been linked to fluconazole resistance in C. glabrata (10). For example, azole resistance has been associated with the overexpression of the drug:H^+^ antiporters (DHA) CgQdr2, CgTpo1_1, CgTpo1_2, and CgTpo3 (11–14). In the case of posaconazole, a study of seven posaconazole-resistant Candida albicans isolates revealed no changes in the expression of the drug transporters Cdr1, Cdr2, and Mdr1 (15), suggesting that posaconazole resistance may be dissociated from antifungal transport.

In many C. albicans clinical isolates, azole resistance arises from point mutations that lead to conformational changes in Erg11, the primary target of azoles (16). In C. glabrata, a G944A mutation in the *ERG11* gene was associated with fluconazole, voriconazole, and polyene resistance in one specific isolate (17). A second fluconazole-resistant isolate of C. glabrata was revealed to have increased expression of *ERG11* due to duplication of the entire chromosome containing this gene (18). However, in all other studies on azole-resistant clinical isolates of C. glabrata, no mutation or upregulation of the *ERG11* gene was observed, suggesting that this is not an important mechanism for clinical acquisition of resistance to azoles (19–21). On the other hand, an E139A mutation in the *ERG3* gene, also involved in ergosterol biosynthesis, was found to lead to increased resistance to fluconazole in C. glabrata strains (22), while in Candida parapsilosis, a similar mutation appeared in a posaconazole-resistant strain (23). Mutation of the *ERG3* gene leads to the formation of ergosta-7,22-dien-3β-ol as the major sterol produced, instead of ergosta-5,7,24(28)-trienol (24). This alteration prevents azole action, since the toxic sterols that accumulate upon the inhibition of Erg11 can no longer be synthesized by this pathway.

Resistance to azole drugs has mostly been examined as a whole, with little distinction between the mechanisms that may be specific to each azole drug. However, several epidemiological surveys on fluconazole, voriconazole, and posaconazole resistance in C. glabrata have revealed that several clinical isolates display different levels of resistance to each of these drugs (25–27). In this work, the azole-susceptible C. glabrata isolate 044, recovered from a positive blood culture, was exposed for prolonged periods to serum-level concentrations of fluconazole resulting in multiazole resistance: after 21 days, posaconazole resistance was reached, followed by clotrimazole resistance after 31 days and, finally, fluconazole and voriconazole resistance upon 45 days of induction. A transcriptomics characterization of the evolution of the 044 clinical isolate from azole susceptibility to stepwise acquisition of resistance to multiple azoles was carried out. On the basis of transcriptomics data, the role of adhesin-encoding genes, especially *CgEPA3*, was investigated in the context of azole drug resistance, establishing a fascinating link between cell-to-cell adhesion, biofilm formation, and drug resistance.

## RESULTS

### Acquisition of resistance in a susceptible C. glabrata clinical isolate.

**FIG 1:**
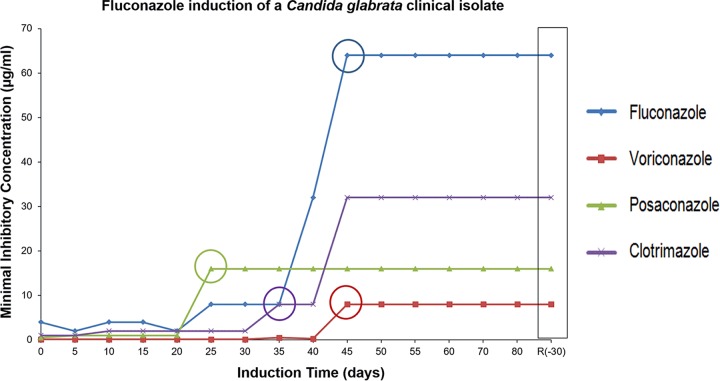
Azole MIC distribution pattern for a *C. glabrata* cell population during evolution toward multiazole resistance. Shown is a comparison of the MICs of fluconazole, voriconazole, posaconazole, and clotrimazole for the 044 *C. glabrata* clinical isolate during prolonged exposure to therapeutic serum fluconazole concentrations (16 µg/ml), as described in Materials and Methods. The time point at which the clinical resistance breakpoint for each azole drug was reached is indicated by a circle.

The 044 clinical isolate was found to exhibit susceptibility to all the azole drugs tested. Specifically, the MIC values obtained for fluconazole, voriconazole, posaconazole, and clotrimazole are 4 µg/ml, 0.25 µg/ml, 0.5 µg/ml, and 0.125 µg/ml, respectively. After exposure to therapeutic serum fluconazole concentrations, the 044 yeast population evolved in a stepwise manner toward multiazole resistance. Specifically, after 21 days, posaconazole resistance was reached (MIC, ≥4 µg/ml), followed by clotrimazole resistance (MIC, ≥4 µg/ml) after 31 days and, finally, fluconazole and voriconazole resistance upon 45 days of induction (MICs, ≥64 and ≥4 µg/ml, respectively) (Fig. 1; Table S1 at http://ibb.tecnico.ulisboa.pt/Cavalheiro_etal_SuplData.pdf). The populations obtained at these time points were designated 044Fluco21 (posaconazole resistant), 044Fluco31 (posaconazole and clotrimazole resistant), and 044Fluco45 (posaconazole, clotrimazole, fluconazole, and voriconazole resistant).

**FIG 2:**
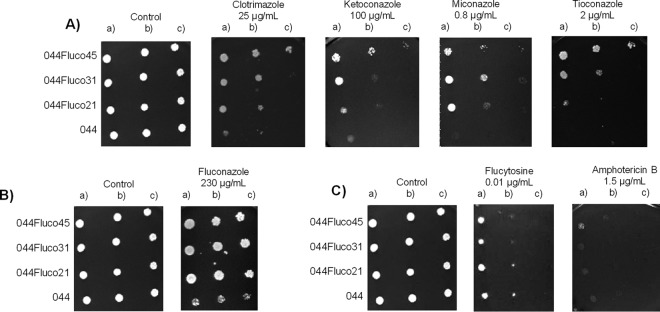
The evolved strains 044Fluco21, 044Fluco31, and 044Fluco45 display increased azole drug tolerance over that of the parental Candida glabrata 044 clinical isolate. Shown are comparisons of the susceptibilities of these strains to the imidazole drugs clotrimazole, ketoconazole, miconazole, and tioconazole (A), the triazole drug fluconazole (B), and amphotericin B and flucytosine (C), at the indicated concentrations, using spot assays on BM agar plates. The inocula were prepared in liquid BM growth medium until the exponential phase of growth was reached, followed by dilution to an OD_600_ of 0.05. Cell suspensions used to prepare the spots were 1:5 (b) and 1:25 (c) dilutions of the cell suspensions used in the plates shown on the left (a). The images displayed are representative of those obtained in at least 3 independent experiments.

By use of spot assays, the 044 clinical isolate and derived populations were further characterized in terms of antifungal drug resistance. This additional assay confirmed the progressive acquisition of resistance to fluconazole and clotrimazole (Fig. 2A and B) and showed that these strains had indeed become multiazole resistant, since increased resistance to ketoconazole, miconazole, and tioconazole was also observed (Fig. 2A). Interestingly, no change in resistance to flucytosine or amphotericin B, two other antifungal drugs of different classes, was observed (Fig. 2C).

### Transcriptional remodeling underlying the stepwise acquisition of resistance to posaconazole, clotrimazole, and fluconazole/voriconazole.

In order to gain insights into the molecular mechanisms underlying the phenotypic changes leading to differential acquisition of resistance to four azoles, the transcriptome-wide changes occurring on the 21st, 31st, and 45th days of fluconazole induction were analyzed by microarray hybridization. It should be noted that the design of the microarrays used was based on the available CBS138 genome; thus, it is possible that specific characteristics of the transcriptome of the clinical isolate analyzed may have been missed in this study. To make sure that the transcriptome observations reflected the evolving population, and not just a specific isolate, a large number of colonies were mixed and analyzed as a whole. Total RNA was extracted from cell populations growing exponentially in YPD medium in the absence of fluconazole in order to ensure that the transcriptome changes observed corresponded to stable transcriptional modifications. Each sample was compared to the azole-susceptible C. glabrata 044 clinical isolate (control), considering a 1.5-fold threshold, with an associated *P* value of <0.05. Overall, the expression of 355 genes was downregulated, whereas the expression of 299 genes was upregulated, in the posaconazole-resistant population obtained after 21 days of fluconazole induction (Tables S2 and S3 at http://ibb.tecnico.ulisboa.pt/Cavalheiro_etal_SuplData.pdf). At day 31, when the population had further acquired clotrimazole resistance, the expression of 73 genes was downregulated, whereas that of 199 genes was found to be upregulated, relative to expression in the parental clinical isolate (Tables S4 and S5). Finally, at day 45, upon the acquisition of fluconazole/voriconazole resistance, the expression of 6 genes was downregulated, whereas that of 27 genes was found to be upregulated, relative to expression in the parental clinical isolate (Tables S6 and S8). There is very little overlap between the differentially expressed genes in the different populations, with only 44 genes whose altered expression was maintained from day 21 to day 31, and 9 upregulated genes on day 31 whose activated expression was maintained until day 45 (Fig. S1 at http://ibb.tecnico.ulisboa.pt/Cavalheiro_etal_SuplData.pdf). GOToolBox was used to identify the Gene Ontology (GO) biological-process terms overrepresented in each data set (Fig. S2).

### Acquisition of azole drug resistance is accompanied by decreased azole drug accumulation.

**FIG 3:**
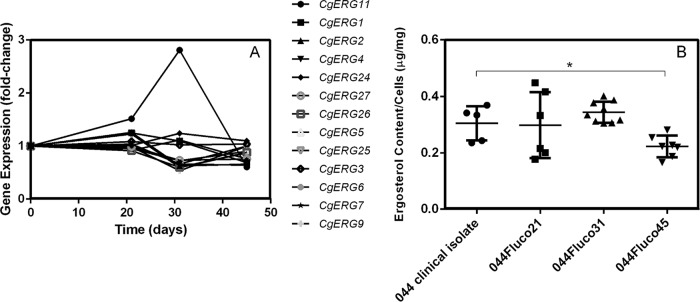
Role of ergosterol metabolism in the acquisition of azole resistance in the 044 clinical isolate. (A) Gene expression changes registered for ergosterol biosynthetic genes along the evolution of the 044 clinical isolate toward multiazole resistance. Transcript levels were obtained by microarray hybridization, and for each, the fold change relative to the level registered for the 044 parental clinical isolate is shown. Values are averages of results from at least three independent experiments. *P* < 0.05. (B) Total ergosterol contents of 044 and azole-derived C. glabrata cells. Cells were harvested after 15 h of growth in YPD medium, and total ergosterol was extracted and quantified by HPLC. Cholesterol was used as an internal standard in order to evaluate the yield of the ergosterol extraction. The ergosterol contents displayed are averages of the results of at least three independent experiments. Error bars represent standard deviations. *, *P* < 0.05.

Two of the most typical changes that occur in azole-resistant strains are alterations in the concentration of ergosterol (28–30) and the activation of drug efflux pumps, leading to reduced accumulation of the drug. Indeed, in the strains that evolved from clinical isolate 044, changes at the level of the expression of ergosterol biosynthetic genes were observed, especially in the case of *ERG11*, whose expression was almost 3-fold higher in the 044Fluco31 strain than in the parental strain but returned to basal levels in the 044Fluco45 strain (Fig. 3A). The total ergosterol concentrations in the susceptible clinical isolate 044 and the derived 044Fluco21, 044Fluco31, and 044Fluco45 cells were therefore determined; they were found to remain constant in the 044Fluco21 and 044Fluco31 cells and to decrease slightly in the 044Fluco45 strain (Fig. 3B). No change in the sequence of the *ERG11* gene was identified in the four strains studied.

**FIG 4:**
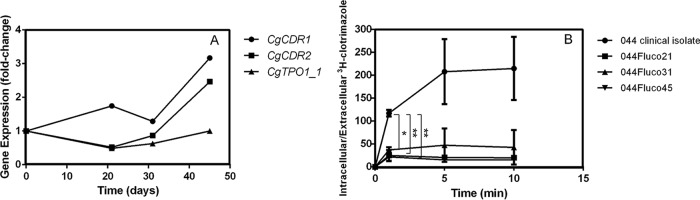
Role of drug export in the acquisition of azole resistance in the 044 clinical isolate. (A) Gene expression changes registered for the multidrug transporter-encoding genes, whose expression reached >2-fold differences, along the evolution of the 044 clinical isolate toward multiazole resistance. Transcript levels were obtained by microarray hybridization, and for each, the fold change relative to the level registered for the 044 parental clinical isolate is shown. Values are averages of results from at least three independent experiments. *P* < 0.05. (B) Time course accumulation of rabiolabeled [^3^H]clotrimazole in strain 044 and the derived strains 044Fluco21, 044Fluco31, and 044Fluco45 during cultivation in liquid BM medium in the presence of 30 mg/liter unlabeled clotrimazole. Accumulation values are the averages of results from at least three independent experiments. Error bars represent standard deviations. *, *P* < 0.05; **, *P* < 0.01.

The expression levels of the multidrug transporter genes *CDR1* and *CDR2* were increased >2-fold only in 044Fluco45 cells (Fig. 4A). This is consistent with the identification of a nonsynonymous point mutation in the sequence of the transcription factor gene *PDR1*. This point mutation, leading to a Y372C substitution, is in the same position as the Pdr1 Y372N gain-of-function (GOF) mutation identified in other azole-resistant isolates of C. glabrata (31) and is similar to that mutation. It is indeed likely that this Y372C substitution constitutes a new Pdr1 GOF mutation, since Pdr1 was found to control nearly 50% of the upregulated genes in this population in cells growing exponentially in the absence of fluconazole or any other stress. Indeed, in an attempt to identify the transcription factors (TFs) that underlie the observed transcriptome-wide remodeling, the PathoYeastract database (32) was used (Table 1). In this search, the PathoYeastract data used were based solely on data published specifically for C. glabrata, demonstrating experimentally the regulatory association between the transcription factors and their target genes in this yeast. The regulators of the expression of the majority of the genes upregulated during the acquisition of multiazole resistance are unknown. The TF that controls the highest number of upregulated genes is Hal9, a TF of unknown function, for the posaconazole-resistant strain and Pdr1 for the remaining two evolved strains.

**TABLE 1:** Distribution of the transcription factors predicted to regulate the genes upregulated in the evolved strains relative to expression in the 044 clinical isolate^*a*^

Strain	TF	% of regulated genes
044Fluco21 (posaconazole resistant)	Hal9	10.51
	**Pdr1**	**4.07**
	CAGL0G08844g	2.71
	Ace2	2.37
	Yap1	2.37
	Skn7	0.68
	Yap5	0.68
	Yap6	0.68
044Fluco31 (clotrimazole/posaconazole resistant)	**Pdr1**	**6.77**
	Yap1	4.69
	Hal9	4.17
	CAGL0G08844g	1.56
	Yap5	1.04
	Yap6	1.04
	Upc2a	0.52
	Skn7	0.52
044Fluco45 (multiazole resistant)	**Pdr1**	**43.75**
	Yap1	18.75
	CAGL0G08844g	6.25
	Skn7	6.25

a The transcription factors (TFs) are listed in the order of the percentage of upregulated genes for each strain, based on the information gathered in the PathoYeastract database, filtered to consider only transcriptional associations known to occur under stress (32). The different rankings of Pdr1 are shown in boldface.

The ability of the evolved cells to reduce the intracellular accumulation of azole drugs was then evaluated using [^3^H]clotrimazole as a model azole drug. Remarkably, the intracellular accumulation of [^3^H]clotrimazole decreased in all three resistant cell populations (Fig. 4B). Specifically, the 044Fluco21 cell population was found to accumulate 4 times less clotrimazole than the 044 parental clinical isolate. The 044Fluco31 and 044Fluco45 cells exhibited even lower levels of accumulated clotrimazole, reaching levels nearly 10-fold-lower than those registered in the 044 clinical isolate (Fig. 4B). Although reduced drug accumulation is consistent with the expression profile of the multiazole-resistant strain 044Fluco45, no clear changes in the expression of drug-resistant transporters were observed in the 044Fluco21 and 044Fluco31 intermediate strains (Fig. 4A), and no change in the sequence of the *PDR1* gene was observed.

### Acquisition of clotrimazole drug resistance is accompanied by increased adhesion.

**FIG 5:**
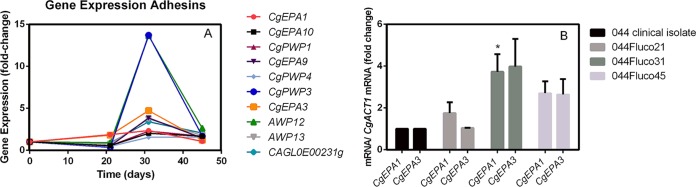
Correlation between adhesin gene expression and the acquisition of clotrimazole/posaconazole resistance in the 044Fluco31 strain. (A) Gene expression changes registered for adhesin-encoding genes along the evolution of the 044 clinical isolate toward multiazole resistance. Transcript levels were obtained by microarray hybridization, and for each, the fold change relative to the level registered for the 044 parental clinical isolate is shown. Values are averages of results from at least three independent experiments. *P* < 0.05. (B) Comparison of the differences in the *CgEPA1* and *CgEPA3* transcript levels along the evolution of the 044 clinical isolate toward multiazole resistance. Transcript levels were obtained by quantitative RT-PCR and were normalized to *CgACT1* mRNA levels. The fold change in the level of each transcript for each evolved strain relative to the level registered for 044 parental cells is shown. Values are averages of results from at least three independent experiments. Error bars represent standard deviations. *, *P* < 0.05.

The expression of adhesin-encoding genes, including the epithelial adhesins Epa1, Epa3, Epa9, and Epa10 and the putative adhesins Awp12, Awp13, Pwp1, Pwp3, Pwp4, and CAGL0E00231g (Fig. 5A), was increased. The transcript levels of the *CgEPA1* and *CgEPA3* genes were verified using quantitative reverse transcription-PCR (RT-qPCR), confirming their transient upregulation, which reached maximal levels in the 044Fluco31 population (Fig. 5B).

**FIG 6:**
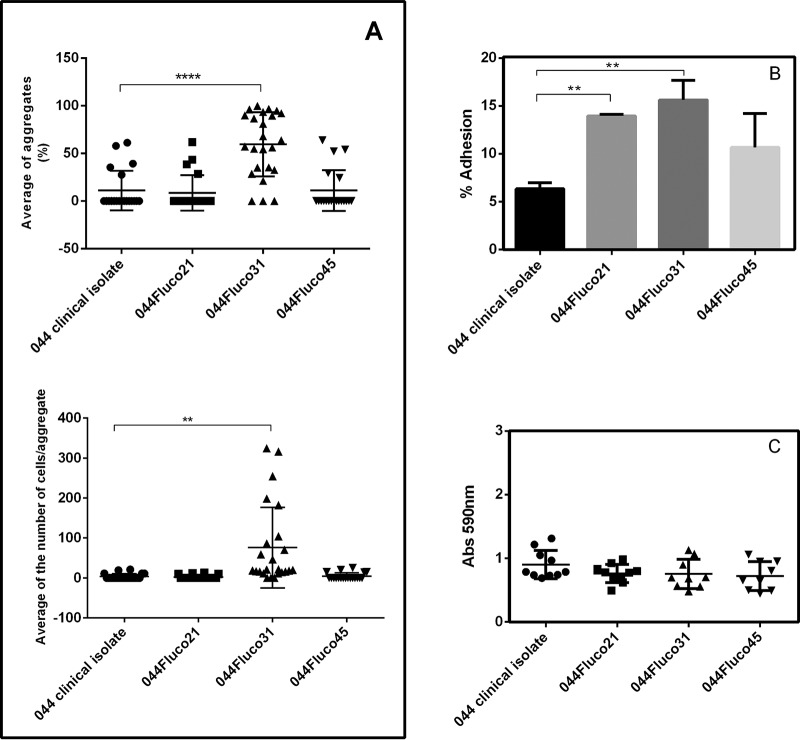
The evolved strain 044Fluco31 exhibits increased cell-to-cell adhesion over that of the 044 clinical isolate. Cell-to-cell aggregation was evaluated based on microscopic observation. (A) Displayed, as scatter dot plots, are the percentage of cell aggregates per total cell population and the number of cells per aggregate, considering aggregates of at least 10 cells. **, *P* < 0.01; ****, *P* < 0.0001. (B) Adhesion of the C. glabrata 044 clinical isolate and derived strains evolving toward azole resistance to VK2/E6E7 human vaginal epithelial cells for 30 min at 37°C under 5% CO_2_. Values are averages of results from at least three independent experiments. Error bars represent standard deviations. **, *P* < 0.01. (C) Biofilm formation on polystyrene surfaces was assessed based on crystal violet staining of the C. glabrata 044 clinical isolate and derived strains evolving toward azole resistance, which had been grown for 15 h in RPMI medium, pH 4.0, in microtiter plates. The data are displayed in a scatter dot plot, where each dot represents the level of biofilm formed in a sample. Horizontal lines indicate the average levels from at least 8 independent experiments. Error bars indicate standard deviations.

This observation prompted us to test the ability of the strains in which azole resistance had evolved to adhere and form biofilms. Indeed, 044Fluco31 cells were found by bright-field microscopy to exhibit a significantly higher level of cell aggregates than 044 and 044Fluco21 cells. The percentage of 044Fluco31 cells found to constitute cell aggregates, considering at least 10 cells/aggregate, reached an average of >50%, in contrast to only 10% for the remaining cell populations (Fig. 6A). Additionally, the number of cells per aggregate, determined by microscopy, was found to be consistently higher for the 044Fluco31 strain (Fig. 6A). Moreover, 044Fluco31 exhibited higher adherence to human vaginal epithelial cells than the other strains (Fig. 6B). Interestingly, the evolved strain 044Fluco21 also exhibited moderately increased adhesion to the epithelial cells tested, despite the observation that there was no clear upregulation of adhesin-encoding genes in this population (Fig. 6B). This suggests that alternative mechanisms whose nature is unclear mediate increased adhesiveness in these C. glabrata cells. Since no differences in biofilm formation on polystyrene dishes was observed (Fig. 6C), the increased expression of adhesin-encoding genes in 044Fluco31 cells appears to correlate with their ability to display cell-to-cell adherence and to adhere to epithelial cells.

### The CgEpa3 adhesin is a new determinant of azole drug resistance.

**FIG 7:**
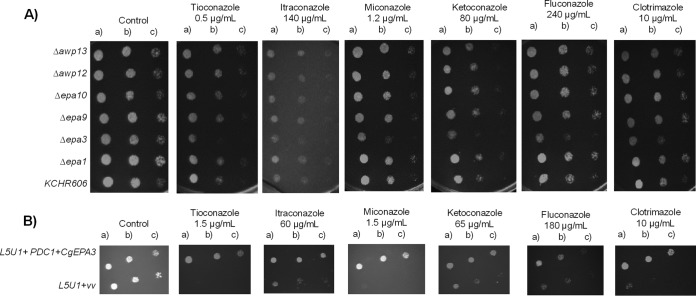
CgEpa3 confers resistance to azole antifungal drugs in C. glabrata cells. (A) Comparison of the susceptibilities of the C. glabrata parental strain KUE100 and the *Δcgepa1*, *Δcgepa3*, *Δcgepa9*, *Δcgepa10*, *Δcgawp12*, *Δcgawp13* derived strains to the imidazole drugs clotrimazole, ketoconazole, miconazole, and tioconazole and the triazole drugs fluconazole and itraconazole at the indicated concentrations by use of spot assays on BM agar plates. (B) Comparison of the susceptibilities to several antifungal drugs, at the indicated concentrations, of the C. glabrata L5U1 strain harboring the pGREG576 cloning vector (vv) and the same strain harboring the pGREG576_*PDC1*_*CgEPA3* plasmids in BM agar plates without uracil by use of spot assays. The inocula were prepared as described in Materials and Methods. The cell suspensions used to prepare the spots were 1:5 (b) and 1:25 (c) dilutions of the cell suspension used in the plates shown on the left (a). The images displayed are representative of at least three independent experiments.

**FIG 8:**
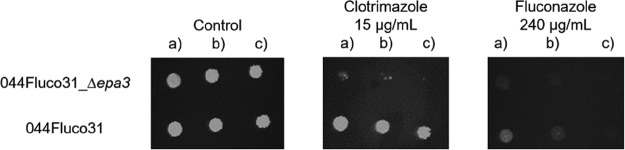
CgEpa3 confers resistance to azole antifungal drugs in the C. glabrata evolved clinical isolate 044Fluco31. Shown is a comparison of the susceptibilities of the 044Fluco31 C. glabrata strain, and the derived 044Fluco31_*Δcgepa3* deletion mutant strain to the azole drugs clotrimazole and fluconazole, at the indicated concentrations, determined by use of spot assays on BM agar plates. The inocula were prepared as described in Materials and Methods. The cell suspensions used to prepare the spots were 1:5 (b) and 1:25 (c) dilutions of the cell suspension used in the plates shown on the left (a). The images displayed are representative of at least three independent experiments.

Given the observation that a high level of expression of adhesin-encoding genes correlates with increased azole resistance in the evolved strain 044Fluco31, the possible role in azole drug resistance of *CgEPA1*, *CgEPA3*, *CgEPA9*, *CgEPA10*, *CgAWP12*, and *CgAWP13*, accounting for most of the more highly upregulated adhesin-encoding genes, was assessed. Deletion of the *CgEPA3* gene, but not the remaining adhesin-encoding genes, increased the susceptibility of C. glabrata to miconazole, ketoconazole, tioconazole, clotrimazole, and fluconazole (Fig. 7A). These results were confirmed by standard assessment of the fluconazole and clotrimazole MICs for the wild-type strain (16 and 2 mg/liter, respectively) and the *Δcgepa3* deletion mutant strain (8 and 1 mg/liter, respectively). Overexpression of *CgEPA3* in the wild-type strain L5U1 was consistently found to increase C. glabrata resistance to miconazole, ketoconazole, tioconazole, clotrimazole, and fluconazole, confirming the role of this gene as a determinant of azole drug resistance (Fig. 7B). To ensure that the deletion of *CgEPA3* also influences drug resistance in the 044Fluco31 background, the effect of its deletion on azole resistance was tested. Deletion of *CgEPA3* was found to increase the susceptibility of the 044Fluco31 strain to clotrimazole and fluconazole (Fig. 8), suggesting that the effect of this adhesin is not strain dependent.

**FIG 9:**
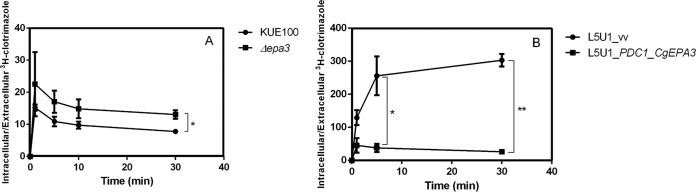
CgEpa3 leads to decreased intracellular accumulation of [^3^H]clotrimazole in C. glabrata cells. Shown are time courses of accumulation of rabiolabeled [^3^H]clotrimazole in the wild-type strain KUE100 and KUE100_*Δcgepa3* (A) and in strain L5U1 harboring the pGREG576 cloning vector (vv) or the pGREG576_*PDC1_CgEPA3* vector (B) during cultivation in liquid BM medium in the presence of 30 mg/liter unlabeled clotrimazole. Accumulation values are the averages of results from at least three independent experiments. Error bars represent standard deviations. *, *P* < 0.05; **, *P* < 0.01.

Since C. glabrata CgEpa3 was identified as conferring resistance to azole drugs, its possible involvement in reducing clotrimazole accumulation in yeast cells was examined. The accumulation of radiolabeled clotrimazole in nonadapted C. glabrata cells suddenly exposed to 30 mg/liter clotrimazole was approximately 2 times higher in cells devoid of CgEpa3 than in wild-type KUE100 cells (Fig. 9A). Overexpression of *CgEPA3* in the wild-type strain L5U1 was consistently found to decrease the accumulation of radiolabeled clotrimazole (Fig. 9B). These findings strongly suggest that CgEpa3 contributes to C. glabrata resistance to azole drugs by reducing their accumulation within yeast cells.

**FIG 10:**
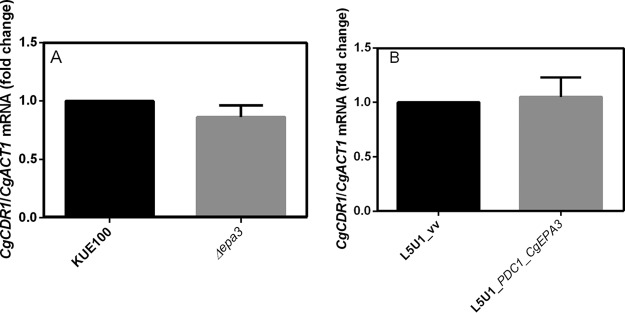
*CgEPA3* expression does not affect *CgCDR1* transcript levels. Shown are transcript levels of *CgCDR1* in the *C. glabrata* wild-type strain KUE100 and KUE100_*Δcgepa3* (A) and in strain L5U1 harboring the pGREG576 cloning vector (vv) or the pGREG576_*PDC1_CgEPA3* vector (B) during cultivation in liquid BM medium. Transcript levels were assessed by quantitative RT-PCR, as described in Materials and Methods. Values are averages of results from at least three independent experiments. Error bars represent standard deviations.

To exclude the possibility that the results observed were due to an indirect effect of *CgEPA3* expression on the expression of the drug efflux pump CgCdr1, the effect of deletion or overexpression of *CgEPA3* on the levels of *CgCDR1* transcripts was evaluated. Interestingly, the expression of *CgEPA3* was found to have no effect on *CgCDR1* transcript levels (Fig. 10), suggesting that CgEpa3 affects azole drug accumulation independently of drug efflux pump activity.

### The CgEpa3 adhesin promotes biofilm formation.

**FIG 11:**
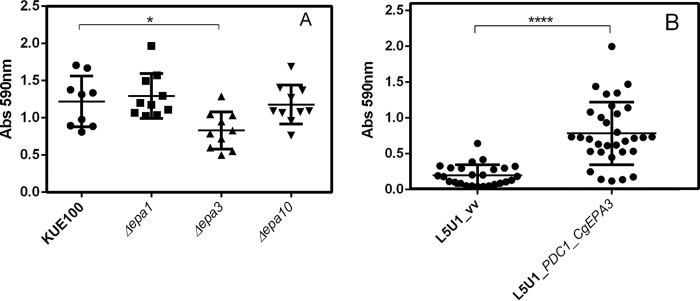
CgEpa3 is required for biofilm formation. (A) Biofilm formation was assessed based on crystal violet staining of cells of wild-type C. glabrata KUE100 and the indicated single deletion mutants, which had been grown for 15 h in SDB medium, pH 5.6, in microtiter plates. The data are displayed on a scatter dot plot, where each dot represents the level of biofilm formed in a sample. Horizontal lines indicate the average levels of biofilm formed in at least 8 independent experiments. Error bars represent standard deviations. *, *P* < 0.05. (B) Biofilm formation on polystyrene surfaces was assessed based on crystal violet staining of wild-type C. glabrata L5U1 cells harboring either the cloning vector pGREG576 (control) (vv) or the pGREG576_*PDC1*_*CgEPA3* plasmid, which had been grown for 24 h in SDB medium, pH 5.6, in microtiter plates. The data are displayed on a scatter dot plot, where each dot represents the level of biofilm formed in a sample. Horizontal lines indicate the average levels of biofilm formed in at least 8 independent experiments. Error bars represent standard deviations. ****, *P* < 0.0001.

The effect of deleting *CgEPA3*, *CgEPA1*, or *CgEPA10* on *in vitro* biofilm formation on 96-well polystyrene microplates was further assessed. Deletion of *CgEPA3*, but not deletion of *CgEPA1* or *CgEPA10*, resulted in an almost 2-fold decrease in the level of total biofilms formed relative to that for the parental strain (Fig. 11A). The overexpression of *CgEPA3* in the wild-type L5U1 strain was consistently found to increase the ability of C. glabrata to form biofilms on polystyrene (Fig. 11B).

## DISCUSSION

**FIG 12:**
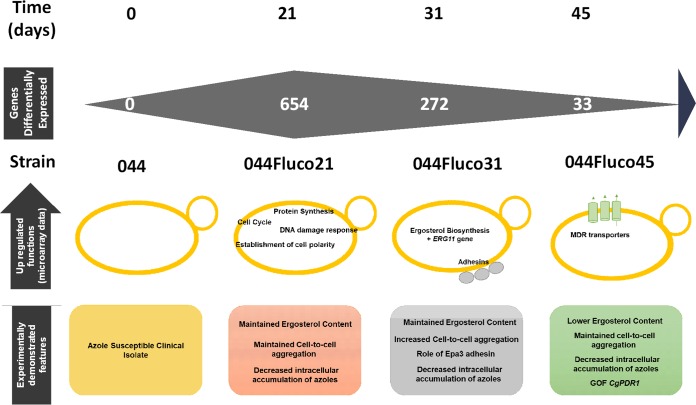
Current model for the mechanisms underlying the evolution of the 044 clinical isolate toward multiazole resistance. Under the timeline of prolonged fluconazole exposure, indicating the days at which resistance to each azole drug was achieved, the main biological processes found to be upregulated in each of the azole-resistant strains are highlighted. Below, the conclusions of the experimental results obtained are given, suggesting that while for 044Fluco21, drug resistance appears to rely mostly on decreased drug accumulation due to increased CgCdr1 expression, the 044Fluco31 strain displays increased drug tolerance due to increased cell-to-cell adhesion, and 044Fluco45 exhibits multiazole resistance due to the acquisition of a CgPdr1 GOF mutation leading to increased expression of CgCdr1 and CgCdr2.

In this study, a transcriptomics analysis of the evolution of an azole-susceptible clinical isolate toward azole resistance, induced by long-standing incubation with a therapeutic concentration of fluconazole in serum, was carried out. The selected approach enabled the identification of the changes in gene expression occurring with time during 45 days of evolution toward a simple and stable solution. One of the surprising observations is that the number of differentially expressed genes, relative to expression in the initial azole-susceptible strain, decreases with time of evolution. At day 21, when the cells were resistant to posaconazole only, 654 genes were found to be expressed differently, while that number decreased to 272 after 31 days and to just 33 at day 45, when the cells reached multiazole resistance. The evolved azole-resistant populations at each time point were found to display significant differences in the molecular mechanisms that are set in place to develop resistance, as summarized in Fig. 12. The multiazole-resistant strain 044Fluco45 exhibits the upregulation of genes encoding multidrug resistance (MDR) transporters *CgCDR1*, *CgCDR2*, and *CgTPO1_2*, decreased accumulation of azole drugs, and a likely gain-of-function (GOF) mutation in the multidrug transcription factor Pdr1. Although the point mutation observed in the *PDR1* sequence, leading to a Y372C substitution, has not been described before, it is indeed very likely to constitute a GOF mutation, given that it is in the same position as the Pdr1 Y372N GOF mutation identified previously (31). Altogether, the results for strain 044Fluco45 are consistent with the recurrent observation that the development of GOF mutations in Pdr1, leading to the upregulation of drug efflux pumps, is the main mechanism of azole resistance acquisition in clinical isolates (8, 9). Based on the evolutionary path observed in the transcriptomics analysis, our current model is that the fluconazole-exposed population appears to be iteratively selected toward resistance at minimum cost, which appears to be, in the long term, the acquisition of Pdr1 GOF mutations, associated with drug efflux pump overexpression. Before reaching that optimal solution, the population goes through transcriptome-wide remodeling, likely reflecting the transient selection of more-fit subpopulations. When the Pdr1 GOF solution is reached by part of the population, these optimized cells are selected, leading to the dilution of other subpopulations until their disappearence.

Interestingly, the 044Fluco21 and 044Fluco31 populations do not exhibit the typical molecular mechanisms related to azole resistance in C. glabrata but still exhibit increased MICs for all azole drugs, resistance to posaconazole and to both posaconazole and clotrimazole, respectively, and increased ability to limit the intracellular accumulation of [^3^H]clotrimazole, compared to the 044 clinical isolate. In the 044Fluco21 posaconazole-resistant strain, the expression of cellular processes such as protein synthesis, cell cycle, and DNA damage response, which are apparently unrelated to azole resistance, is upregulated. Thus, although the slight upregulation of *CgCDR1* in the 044Fluco21 strain may, at least partially, account for the posaconazole resistance phenotype, further characterization of the mechanisms of posaconazole resistance acquisition in C. glabrata are required.

The 044Fluco31 strain was found to display upregulation of the *ERG11* gene; however, the concentration of ergosterol was found to remain constant in this strain. Assuming that the increased *ERG11* gene expression results in increased Erg11 protein expression, this may at least prevent the decrease in the ergosterol content that fluconazole exposure is bound to induce and may thus decrease azole susceptibility by maintaining the Erg11/drug molecule ratio. Although upregulation of the *ERG11* gene (33, 34) and the augmentation of ergosterol levels (30) are associated with azole resistance in Candida albicans, in the case of C. glabrata, the expression level or amino acid substitutions of the *ERG11* gene do not seem to correlate with azole resistance acquisition in the clinical setting (19, 20, 35). Given that there is no corresponding increase in ergosterol levels (as observed), the increased expression of *ERG11* may only partially, not completely, explain the observed gain in azole resistance in strain 044Fluco31. This observation prompted us to analyze in more detail the molecular basis underlying the posaconazole and clotrimazole resistance exhibited by strains 044Fluco31. Strain 044Fluco31 was found to exhibit upregulation of several adhesin-encoding genes, accompanied by an increased ability to adhere to other C. glabrata cells and to epithelial cells. Among the adhesin-encoding genes upregulated in the 044Fluco31 strain, we focused our research on three adhesins of the EPA family, encoded by the *CgEPA1*, *CgEPA3*, and *CgEPA10* genes. Significantly, the expression of Epa3 was found to decrease C. glabrata susceptibility to azole drugs, directly or indirectly leading to decreased accumulation of azole drugs. These results indicate Epa3 as an important, though unexpected, player in azole resistance. Our current model is that the role of CgEpa3, and possibly that of other adhesins, in azole resistance, might be to protect the cells from the extracellular concentration of the drug by promoting cell aggregation. Interestingly, comparing the genome of an azole-susceptible C. glabrata isolate with that of an azole-resistant C. glabrata isolate showed a higher number of adhesin-like genes in the resistant isolate (36). As expected, CgEpa3 was also found to play a role in C. glabrata adhesion and biofilm formation, a finding consistent with the predicted role of CgEpa3 and its upregulation in C. glabrata biofilms *in vitro* (37).

Altogether, the analysis of the evolution toward multiazole resistance of the 044 clinical isolate suggests that prolonged exposure to fluconazole progressively selects the subpopulation that evolves to higher resistance with lower costs, leading to what appears to be a unique response to fluconazole induction. Indeed, the final transcriptional profile reached by the 044Fluco45 strain gives evidence of the important role of Pdr1 GOF mutations and the activation of MDR transporters in this context. Nevertheless, in the path to full resistance, several other, eventually more subtle, mechanisms of azole resistance may be employed by the evolving population, including the overexpression of adhesin-encoding genes. This study highlights the role of one of these genes, CgEpa3, in azole drug resistance, further supporting the notion that azole resistance is a multifactorial process, composed of different molecular mechanisms that should be considered in the design of better-suited therapeutic strategies.

## MATERIALS AND METHODS

### Strains and growth medium.

The 044 clinical isolate of Candida glabrata studied here was collected from a patient attending the Centro Hospitalar de São João in Porto, Portugal. C. glabrata strains 044Fluco21 (pozaconazole resistant), 044Fluco31 (resistant to pozaconazole and clotrimazole), and 044Fluco45 (resistant to pozaconazole, clotrimazole, fluconazole, and voriconazole) were obtained in this study through the directed evolution of the 044 clinical isolate, as described bellow. Additionally, the wild-type KUE100 and CBS138 C. glabrata strains were used. Cells were batch-cultured at 30°C with orbital agitation (250 rpm) in the following growth media: yeast extract-peptone-dextrose (YPD) growth medium, containing, per liter, 20 g glucose (Merck), 20 g yeast extract (Difco), and 10 g bacterial peptone (LioChem); BM minimal growth medium, containing, per liter, 20 g glucose (Merck), 2.7 g (NH_4_)_2_SO_4_ (Merck), and 1.7 g yeast nitrogen base without amino acids or (NH_4_)_2_SO_4_ (Difco); Roswell Park Memorial Institute (RPMI) 1640 medium, containing 18 g glucose (Merck), 10.4 g RPMI 1640 (Sigma), and 34.53 g morpholinepropanesulfonic acid (MOPS; Sigma) per liter; and Sabouraud’s dextrose broth (SDB), containing 40 g glucose (Merck) and 10 g peptone (LioChem) per liter.

The VK2/E6E7 human epithelial cell line (ATCC CRL-2616) was used for adhesion assays. This cell line is derived from the vaginal mucosa of a healthy premenopausal female subjected to vaginal repair surgery and was immortalized with human papillomavirus 16/E6E7. Cells maintenance was achieved with keratinocyte–serum-free medium, containing 0.1 ng/ml human recombinant epidermal growth factor (EGF), 0.05 mg/ml bovine pituitary extract, and an additional 44.1 mg/liter calcium chloride. Cells were maintained at 37°C, with 95% air and 5% CO_2_.

### *In vitro* induction of multiple azole resistance.

Three randomly selected colonies of the 044 clinical isolate, exhibiting susceptibility to all azoles tested, was incubated in 10 ml of YPD medium overnight in a rotating drum at 150 rpm and 35°C. A 1-ml aliquot of this culture, containing 10^6^ blastoconidia, was transferred to different vials, each containing 9 ml of culture medium with or without 16 mg/ml fluconazole, a concentration of the drug that corresponds to therapeutic levels in serum obtained during antifungal treatment (38), and was incubated overnight as described above. The following day, aliquots from each culture containing 10^6^ blastoconidia were again transferred to fresh medium containing the same antifungal and were reincubated as described above. Each day, for the 80 days of the assay, a 1-ml aliquot from each subculture was mixed with 0.5 ml of 40% glycerol and was frozen at –70°C for later testing. To assess resistance stability, the resistant isolates obtained were subcultured daily in the absence of the drug for 30 days. Three colonies from each isolate were incubated in 10 ml drug-free YPD medium at 35°C and 150 rpm. The following day, aliquots were transferred to fresh medium. At each subculture, a 1-ml aliquot of the suspension was mixed with 0.5 ml of 40% glycerol, and the mixture was frozen at –70°C for further testing.

### Drug susceptibility assays.

The MIC values of each antifungal drug were determined according to the M27-A3 protocol and the M27-S4 supplement of the Clinical and Laboratory Standards Institute (CLSI) (39). Interpretative criteria for fluconazole and voriconazole were those of the CLSI: for fluconazole, a susceptible–dose-dependent (S-DD) MIC of ≤32 mg/ml and a resistance (R) MIC of ≥64 mg/ml; for voriconazole, a wild-type MIC of ≤0.5 mg/ml and a non-wild-type MIC of ≥1 mg/ml. Although susceptibility breakpoints have not yet been established for posaconazole or clotrimazole, strains inhibited by ≤4 mg/ml were considered to be susceptible to posaconazole or clotrimazole, respectively, considering that their breakpoints should be 4-fold higher in C. glabrata than in C. albicans, as is the case for fluconazole (40, 41). Every 5 days of incubation, with or without the antifungal, MIC values were redetermined for fluconazole, voriconazole, posaconazole, and clotrimazole. Candida glabrata type strain CBS138 was used in each testing assay, as recommended.

The antifungal drug susceptibilities of the 044 clinical isolate and the derived azole-resistant strains (1), of the KUE100 parental strain and the derived *Δcgepa1*, *Δcgepa3*, and *Δcgepa10* deletion mutants (2), and of the C. glabrata wild-type strain L5U1 harboring the pGREG576 vector or the pGREG576_*PDC1*_*CgEPA3* plasmids (3) were evaluated by spot assays, as described previously (11).

### Transcriptomic analysis.

The C. glabrata 044 clinical isolate and the derived 044Fluco21 (pozaconazole-resistant), 044Fluco31 (pozaconazole- and clotrimazole-resistant), and 044Fluco45 (pozaconazole-, clotrimazole-, fluconazole-, and voriconazole-resistant) strains were harvested in the mid-exponential phase of growth in YPD medium. Three independent cultures from each strain were used for transcriptional profiling. RNA extraction was performed as described elsewhere (42). The quality and integrity of the purified RNA were confirmed using a bioanalyzer. The DNA chips used for this microarray analysis were manufactured by Agilent using a design for C. glabrata (43). The microarray was designed using eArray by Agilent Technologies, based on the annotation of C. glabrata CBS138 available at the Yeast Gene Order Browser in 2014 (44). cDNA synthesis, hybridization, and scanning were performed using protocols similar to those described in reference 43, except that hybridization was carried out using an Agilent hybridization oven at 65°C for 17 h at 100 rpm, according to a previously described protocol (42). Data were analyzed using the LIMMA package in Bioconductor (www.bioconductor.org), as described before (42). Each gene was represented by two probes spotted in duplicate, which were used separately to calculate the log fold change (FC) (Tables S1 to S3 at http://ibb.tecnico.ulisboa.pt/Cavalheiro_etal_SuplData.pdf). Only genes exhibiting a log_2_ FC of >1 and a *P* value of ≤0.05 for at least one probe were selected for further analysis. Gene Ontology enrichment analysis was performed with the GOToolBox Web server (45) for each group of upregulated and downregulated genes, considering C. glabrata genes or their Saccharomyces cerevisiae homologs. Predictive analysis of the transcription factors controlling the observed transcriptional alterations was conducted using the PathoYeastract database (32).

### Cloning of the C. glabrata *CgEPA3* gene (ORF *CAGL0E06688g*).

The pGREG576 plasmid from the Drag&Drop collection (46) was used to clone and express the C. glabrata open reading frame (ORF) *CAGL0E06688g* in S. cerevisiae, as described before for other heterologous genes (47). pGREG576 was acquired from Euroscarf and contains a galactose-inducible promoter (*GAL1*) and the yeast selectable marker *URA3*. The *CgEPA3* gene was cloned in two sections, both generated by PCR, using CBS138 genomic DNA and the primers listed in Table 2. To enable expression of the *EPA3* gene in C. glabrata, the *GAL1* promoter was replaced by the constitutive C. glabrata *PDC1* promoter. The *PDC1* promoter DNA was generated by PCR using the primers listed in Table 2. The recombinant plasmids pGREG576_*CgEPA3* and pGREG576_*PDC1*_*CgEPA3* were obtained through homologous recombination in S. cerevisiae and were verified by DNA sequencing.

**TABLE 2:** List of primers used in this study

Purpose and name	Sequence (3′–5′)
CgEPA3 gene cloning	
* pGREG_CgEPA3_I_FW*	TGCTATTAGGTCAACCTTGTAACAGCTGCCATAGCTATTCGAACTATAGCTTAAG
* pGREG_CgEPA3_I_Rev*	CAGTTGCCACGATGACTAGTCAGCTGGAGCTCAGTACATTAATCAATACAGTGCG
* pGREG_CgEPA3_II_FW*	GTCATCGTGGCAACTGTGATTTTCTTTCTAGATTCCTACT
* pGREG_CgEPA3_II_Rev*	CGTGAAAAAAAGACTAAATTCAGCTGGAGCTCAGTACATTAATCAATACAGTGCG

pGREG576 GAL-to-PDCI promotor replacement	
* pGREG_PDCI_ FW*	TTAACCCTCACTAAAGGGAACAAAAGCTGGAGCTCAGCATTTTTATACACGTTTTAC
* pGREG_PDCI_Rev*	GAAAAGTTCTTCTCCTTTACTCATACTAGTGCGGCTGTTAATGTTTTTTGGCAATTG

CgEPA1, CgEPA3, CgEPA9, CgEPA10, AWP12 and AWP13 gene disruption	
* ΔCgEPA1_FW*	GCACTAGTCGCCGGACAAAATCAAACCAATTAACGTTTCTGATTTAGAGTTCTTACTTCTTTTTCGAAAC
* ΔCgEPA1_Rev*	GGTCGGAGTGCTACATTTTGGTCCTTTATATTATTGAAGGTATCAAATCGTAACATTTTCGACACCACCG
* ΔCgEPA3_FW*	GCACTAGTCGCCGGTAACTAAAAAAAAGAAAACATTGAAAAAGACTAGTTAAATTAAGCTAATACTACCA
* ΔCgEPA3_Rev*	GGTCGGAGTGCTACATTAAGACAAGGGAATTAAAGATAATACTATAACAAAGAGAAATAAGTAACTAATT
* ΔCgEPA3_SAT1_FW*	ATCATTCCTGGTTTGACAATGGAACTTTACTCTTACCCTATTGTCAGTGGTACTCCTCCCATGGACGGTGGTATGTTTTA
* ΔCgEPA3_SAT1_Rev*	GCTTCGAAGCTTTACCCTTGTACTCTGAAAAATTAGGGATATTAAAGACAGAACTAGAGGTTAGGCGTCATCCTGTGCTC
* ΔCgEPA9_FW*	TCACTAAGTGGATGAAATGCAGAAAGAATACAATTTAACCCTCTTCAATGCGATCCGGCGGCCGCTGATCACG
* ΔCgEPA9_Rev*	CAACTGCCAAAGGGTTCACAAGTTGAATACCAGGCCAACCATCATCAGTGGCAGGGCGACATCGTGAGGCTGG
* ΔCgEPA10_FW*	GCACTAGTCGCCGGGCTAATACCTGATCGTAGACTATATTACTTCATAAAACTTTTGTTACCTACCCGTA
* ΔCgEPA10_Rev*	GGGAAAGAAAAATCAACCACATTGGCAAAACGTAGACATCTTGTCAACTGCCAAAGCATCGTGAGGCTGG
* ΔCgAWP12_FW*	TACTTAAAATTTCCTTTCTCAATCAACAAAAATATCCATTAACTGAAAAAGAATACGGCGGCCGCTGATCACG
* ΔCgAWP12_Rev*	TATCAATGTTCATATTGAAAGAAACAGCCTATCTAAAAATCATGGCTGGACGAACGCGACATCGTGAGGCTGG
* ΔCgAWP13_FW*	ATTACGTGACAAAAGACAGATAAAGGAATTCTAAATATCCATCTACAAGACCATTCGGCGGCCGCTGATCACG
* ΔCgAWP13_Rev*	CCTATCTAAAAATCATGGCTGGACGAACGTTTGTATTACTGTACATGTTGGGCATTCGACATCGTGAGGCTGG
* ΔCgEPA1_conf_FW*	TAAATAAGTTTTTATCTCGACC
* ΔCgEPA1_conf_Rev*	GGTTTTCATTGACCGAAG
* ΔCgEPA3_conf_FW*	CGGAACTTCATTGGTATG
* ΔCgEPA3_conf_Rev*	CATACCAATGAAGTTCCG
* ΔCgEPA3_SAT1_conf_FW*	CCCAGAACCCATTTGGTTATCC
* ΔCgEPA3_SAT1_conf_Rev*	GCCCAGATAACAACACAAGTCC
* ΔCgEPA9_conf_FW*	CTTTTGGTATCTGACTCTGTTAT
* ΔCgEPA9_conf_Rev*	GGTCCCAAGAGATTTTACC
* ΔCgEPA10_conf_FW*	TGTCTCAGTCTATGGCTTTC
* ΔCgEPA10_conf_Rev*	CTCAAGTGTCTCCCAACA
* ΔCgAWP12_conf_FW*	GCCTGTGGATATTGCTACT
* ΔCgAWP12_conf_Rev*	GTCACTGATTGAAAGTTCTCG
* ΔCgAWP13_conf_FW*	GTATATTTTCAAGTGCTGCAT
* ΔCgAWP13_conf_Rev*	CTACCGTGGTTTCACTTG

RT-PCR experiments	
* CgEPA1_FW*	TTGATTGCTGCAGAAGGGATT
* CgEPA1_Rev*	ATGGCGTAGGCTTGATAATTTCC
* CgEPA3_FW*	GTTCCTGTCACCAGCACCATATACTA
* CgEPA3_Rev*	CCTTGGCAACTAGGTGTTTGG
* CgCDR1_FW*	GCTTGCCCGCACATTGA
* CgCDR1_Rev*	CCTCAGGCAGAGTGTGTTCTTTC
* CgACT1_FW*	AGAGCCGTCTTCCCTTCCAT
* CgACT1_Rev*	TTGACCCATACCGACCATGA

### Disruption of the C. glabrata *CgEPA1*, *CgEPA3*, *CgEPA9*, *CgEPA10*, *CgAWP12*, and *CgAWP13* (ORF *CAGL0E06644g*, *CAGL0E06688g*, *CAGL0A01366g*, *CAGL0A01284g*, *CAGL0G10219g*, and *CAGL0H10626g*) genes.

The deletion of the *CgEPA1*, *CgEPA3*, *CgEPA9*, *CgEPA10*, *CgAWP12*, and *CgAWP13* genes was carried out in the parental strain KUE100, using the method described in reference 48. The target genes were replaced, through homologous recombination, by a DNA cassette including the *CgHIS3* gene. The pHIS906 plasmid including *CgHIS3* was used as a template, and transformation was performed as described previously (48). The deletion of *CgEPA3* in the 044Fluco31 background was carried out using the method described in reference 49. The gene was replaced through homologous recombination by a *SAT1* flipper cassette. PCR was used to prepare the replacement cassettes and to verify recombination loci and gene deletions, using the primers listed in Table 2.

### Gene expression analysis.

The levels of *CgEPA1* and *CgEPA3* transcripts in the azole-susceptible isolate 044 and the azole-resistant derived strains, as well as the levels of *CgCDR1* transcripts in the wild-type strain KUE100 and its derived *Δcgepa3* deletion mutant strain, and in strain L5U1 harboring the cloning vector pGREG576 or the *CgEPA3* expression plasmid pGREG576_*PDC1_CgEPA3*, were assessed by quantitative real-time PCR. Total-RNA samples were obtained from cell suspensions harvested under control conditions, in the absence of drugs. Synthesis of cDNA for real-time RT-PCR experiments, from total-RNA samples, was performed using the Multiscribe reverse transcriptase kit (Applied Biosystems), following the manufacturer’s instructions, and using 10 ng of cDNA per reaction. The RT-PCR step was carried out using SYBR green reagents. Primers for the amplification of the *CgEPA1*, *CgEPA3*, *CgCDR1*, and *CgACT1* cDNA were designed using Primer Express software (Applied Biosystems) (Table 2). The RT-PCRs were conducted in a thermal cycler block (7500 Real-Time PCR system; Applied Biosystems). The *CgACT1* mRNA level was used as an internal control. The relative values obtained for the wild-type strain under control conditions were set at 1, and the remaining values are presented relative to that control.

### [^3^H]clotrimazole accumulation assays.

[^3^H]clotrimazole transport assays were carried out as described before (11). To estimate the accumulation of clotrimazole (intracellular/extracellular [^3^H]clotrimazole), yeast cells were grown in BM medium until the mid-exponential phase and were harvested by filtration. Cells were washed and resuspended in BM medium to obtain dense cell suspensions (optical density at 600 nm [OD_600_], 5.0 ± 0.2, equivalent to approximately 2.2 mg [dry weight] ml^−1^). After a 5-min incubation at 30°C, with agitation (150 rpm), 0.1 µM [^3^H]clotrimazole (American Radiolabelled Chemicals; 1 mCi/ml) and 30 mg/liter of unlabeled clotrimazole were added to the cell suspensions. The intracellular accumulation of labeled clotrimazole was followed for 30 min, as decribed elsewhere (11). To calculate the intracellular concentration of labeled clotrimazole, the internal cell volume (Vi) of the exponential cells was considered constant and equal to 2.5 µl (mg [dry weight]^−1^) (50).

### Quantification of total cellular ergosterol.

Ergosterol was extracted from cells using the following method adapted from reference 51, as described before (52). Cells were cultivated in YPD medium with orbital agitation (250 rpm) until stationary phase, harvested by centrifugation, and resuspended in methanol. Cholesterol (Sigma) was added as an internal standard to estimate the ergosterol extraction yield. The extracts were analyzed by high-pressure liquid chromatography (HPLC) with a 250-mm by 4-mm C_18_ column (LiChroCART Purospher STAR RP-18, end-capped, 5 mm) at 30°C. Samples were eluted in 100% methanol at a flow rate of 1 ml/min. Ergosterol was detected at 282 nm with a retention time of 12.46 ± 0.24 min, while cholesterol was detected at 210 nm with a retention time of 15.36 ± 0.35 min. Results are expressed in micrograms of ergosterol per milligram (wet weight) of cells.

### Biofilm quantification.

C. glabrata strains were tested for their capacity for biofilm formation by use of the crystal violet method. For that, the C. glabrata strains were grown in SDB medium and harvested by centrifugation at mid-exponential phase. The cells were inoculated with an initial OD_600_ of 0.05 ± 0.005 in 96-well polystyrene microtiter plates (Greiner) in either SDB (pH 5.6) or RPMI (pH 4) medium. Cells were cultivated at 30°C for 15 ± 0.5 h or 4 ± 0.5 h with mild orbital shaking (70 rpm). After the incubation time, each well was washed three times with 200 µl of deionized water to remove cells not attached to the biofilm matrix. Then 200 µl of a 1% crystal violet (Merck) alcoholic solution was used to stain the biofilm present in each well. Following 15 min of incubation with the dye, each well was washed with 250 µl of deionized water. The stained biofilm was eluted in 200 µl of 96% (vol/vol) ethanol, and the absorbance of each well was read in a microplate reader at a wavelength of 590 nm (SPECTROstar Nano; BMG Labtech).

### Human vaginal epithelial cell adherence assay.

For the adhesion assays, VK2/E6E7 human epithelial cells were grown and inoculated in 24-well polystyrene plates (Greiner) with a density of 2.5 × 10^5^ cells/ml a day prior to use. Additionally, C. glabrata cells were inoculated with an initial OD_600_ of 0.05 ± 0.005 and were cultivated at 30°C for 16 ± 0.5 h with orbital shaking (250 rpm) in YPD medium. In order to initiate the assay, the culture medium of mammalian cells was removed and replaced by new culture medium in each well, and, subsequently, Candida glabrata cells were added to each well, with a density of 12.5 × 10^5^ CFU/well, corresponding to a multiplicity of infection (MOI) of 10. Then the plate was incubated at 37°C under 5% CO_2_ for 30 min. Afterwards, each well was washed 3 times with 500 µl of 1× phosphate-buffered saline (PBS) (pH 7.2), followed by the addition of 500 µl of 0.5% Triton X-100 and incubation at room temperature for 15 min. The cell suspension in each well was then recovered, diluted, and spread onto agar plates to determine the CFU count, which represents the proportion of cells adherent to the human epithelium.

### Accession number(s).

The data sets were deposited at the Array Express Database with reference number E-MTAB-6787.
